# Unravelling the Perspectives of Day and Night Traders in Selected Markets within a Sub-Saharan African City with a Malaria Knowledge, Attitude and Practice Survey

**DOI:** 10.3390/ijerph18073468

**Published:** 2021-03-26

**Authors:** Patience B. Tetteh-Quarcoo, Nicholas T. K. D. Dayie, Kevin Kofi Adutwum-Ofosu, John Ahenkorah, Emmanuel Afutu, Seth K. Amponsah, Mubarak Abdul-Rahman, James-Paul Kretchy, Janet Y. Ocloo, Nicholas I. Nii-Trebi, Akua K. Yalley, Oheneba C. K. Hagan, Benjamin P. Niriwa, Chukwuemeka C. Aghasili, Fleischer C. N. Kotey, Eric S. Donkor, Patrick F. Ayeh-Kumi, Emilia Asuquo Udofia

**Affiliations:** 1 Department of Medical Microbiology, University of Ghana Medical School, Accra P.O. Box KB 4236, Ghana; eafutu@ug.edu.gh (E.A.); pullebenjamin@gmail.com (B.P.N.); fcnkotey@flerholiferesearch.com (F.C.N.K.); esampane-donkor@ug.edu.gh (E.S.D.); pfayeh-kumi@ug.edu.gh (P.F.A.-K.); 2 Department of Anatomy, University of Ghana Medical School, Accra P.O. Box KB 4236, Ghana; kadutwum-ofosu@ug.edu.gh (K.K.A.-O.); jahenkorah@ug.edu.gh (J.A.); 3 Department of Medical Pharmacology, University of Ghana Medical School, Accra P.O. Box KB 4236, Ghana; sethicom@yahoo.com; 4 Department of Pathology, University of Ghana Medical School, Accra P.O. Box KB 4236, Ghana; presmubarak@gmail.com; 5 Department of Physician Assitantship Studies/Public Health, School of Medicine and Health Sciences, Central University, Miotso P.O. Box DS 2305, Accra, Ghana; jkretchy@central.edu.gh; 6 Department of Pathology, Korle-Bu Teaching Hospital, Korle-Bu, Accra P.O. Box 77 233, Ghana; janeteocloo@gmail.com; 7 Department of Medical Laboratory Sciences, School of Biomedical and Allied Health Sciences, University of Ghana, Korle Bu, Accra P.O. Box KB 143, Ghana; ninii-trebi@ug.edu.gh (N.I.N.-T.); akyalley@ug.edu.gh (A.K.Y.); 8 Department of Medical Biochemistry, School of Medical Sciences, College of Health and Allied Sciences, University of Cape Coast, Cape Coast, Ghana; ock.hagan@uccsms.edu.gh; 9 Holy Family Hospital, Techiman P.O. Box 36, Ghana; 10 Department of Community Health, University of Ghana Medical School, Accra P.O. Box KB 4236, Ghana; aghasilichristian@gmail.com (C.C.A.); eudofia@ug.edu.gh (E.A.U.); 11 FleRhoLife Research Consult, Teshie, Accra P.O. Box TS 853, Ghana

**Keywords:** perspectives, malaria, knowledge, attitude, practice, market, traders, day, night

## Abstract

Background: Malaria is still endemic in sub-Saharan Africa, with a high disease burden. Misconceptions about malaria contribute to poor attitudes and practices, further increasing the burden in endemic countries. Studies have examined the knowledge, attitudes, and practices (KAP) of malaria among different populations. However, there seems to be no available literature reporting on the perspectives of day and night market traders. To the best of our knowledge, this is the first report on malaria KAP with a focus on day and night market traders. Methods: A descriptive cross-sectional study involving day and night market traders in 10 selected markets within the Greater Accra Region of Ghana was carried out. Data were collected from consenting respondents using a structured questionnaire. Results: Of the 760 respondents (33.3% (*n* = 253) night and 66.7% (*n* = 507) day traders) interviewed, there was no significant difference between the day and night market traders in terms of malaria KAP. Although the market traders had an overall moderate knowledge (54.0% of the day traders and 56.5% of the night traders), misconceptions about malaria (especially that it could be caused by exposure to the sun) still existed among the traders. Moreover, the majority of the traders who demonstrated high knowledge (43.98%, *n* = 250) did not always take laboratory tests to confirm their suspicion, indicating poor attitude. Furthermore, the market traders’ choice of drug for malaria treatment (*p* = 0.001) and preferred malaria treatment type (orthodox or herbal) (*p* = 0.005) were significantly associated with their knowledge level. Conclusions: Despite the observation that no significant difference in KAP exists between day and night market traders, appropriate health education programs and interventions still need to be directed at misconceptions, poor attitudes, and poor practices revealed by this study. This will ultimately help in the prevention and control of malaria in Ghana, and globally.

## 1. Introduction

Malaria is caused by the *Plasmodium* parasite, which is transmitted by the bite of a female anopheles mosquito vector. Vector occurrence is highly dependent on the region and the environment. In sub-Saharan Africa with Ghana inclusive, the *Anopheles gambiae* complex is the most important vector of malaria [1]. Its breeding sites are usually freshwater pools created in the rainy season and potentially as spill-over from dams, which leads to a surge in malaria disease during the rainy season and around dam areas [1]. Increase in malaria can result from a rise in outdoor biting, which can vary between locations depending on endemicity, mosquito species, as well as history of malaria control interventions. Moreover, sociocultural and economic factors, which may include customs, lifestyles and the environment, can be considered vital determinants of malaria transmission [1]. However, because mosquitoes are only able to bite humans at a particular point in time when they both find themselves at the same place, and human activities such as trading at the market during the night may be equally vital drivers of persistent transmission. Most markets in Greater Accra are surrounded by clogged gutters that contain wastewater and pose a risk for the transmission of malaria.

Malaria poses an enormous burden to the world’s population, with most cases occurring in sub-Saharan Africa [1]. According to the 2017 World Malaria Report, progress in malaria control has stalled after the initial unprecedented global success [2]. Malaria is endemic in Ghana [3] and is known as a serious impediment to social and economic development [1].

In 2018, notable increases in malaria cases were observed in Ghana (8%) and Nigeria (5%) [4]. In Ghana, within the first quarter of 2017, about 2.3 million suspected malaria cases were recorded at the out-patient department of one municipal hospital, representing an increase of 1.2% over the same period in 2016 [5]. One major problem facing the prevention and control of malaria in Ghana is delay in health-seeking due to wrong perceptions of the disease [5]. The socio-economic aspects of a population are known to contribute significantly to the epidemiology and control of parasitic diseases [6], such as *Plasmodium* infection. For example, a study to determine sociocultural factors influencing malaria control in North Central Nigeria [7] observed that many of the respondents did not view malaria as a serious problem. The authors further revealed that marriage, educational level, and some occupations appeared to have a positive influence on malaria knowledge [7]. Moreover, low educational levels and poverty have been asserted to influence malaria spread and treatment-seeking behavior [8,9]. A study conducted in India by Sabin et al. [10] revealed that even though the study respondents perceived malaria as a condition of high clinical significance, they still endorsed traditional beliefs and practices, such as the use of unapproved preventive and treatment strategies [10]. These reports underpin the importance of assessing the knowledge, attitudes, and practices (KAP) of individuals regarding malaria so as to provide insights into perceptions and behaviors that could aid in tailoring appropriate health education programs and interventions.

Various studies have been carried out to examine the KAP of malaria among different groups of people. For example, in Ghana, studies on KAP of malaria among the populace focused on different study groups, highlighting different aspects of malaria KAP, such as those of rural and urban communities [11,12,13,14]. Others include KAP of insecticide-treated net use in malaria prevention [15,16] and choice of malaria treatment regimens [17,18]. Some researchers also concentrated their studies on KAP of malaria among healthcare workers [15,17,19,20,21,22] and vulnerable groups such as HIV patients [23]. Elsewhere, there have been reports on malaria KAP among pregnant women in Ethiopia [24], tertiary students in Nigeria [25,26], primary school children in Tanzania [27], and people visiting some referral hospitals in Eritrea [28]. Other community-based KAP studies have been conducted in various locations, such as northern Nigeria [29]; southwestern Saudi Arabia [30]; southwest Ethiopia [31]; municipalities of Tierralta, Buenaventura, and Tumaco in Colombia [32]; and four Lubombo Spatial Development Initiative (LSDI) sentinel sites in Swaziland [33].

One important section of the population that seems to have been overlooked by malaria KAP studies conducted in Ghana is market traders. In Nigeria, one study that touched on malaria KAP of traders [18] contrasted them with artisans, reporting low knowledge on malaria, with the latter group having relatively poorer knowledge. Interestingly, in another study in that same country [8], some traders opined that the sun was the cause of malaria. Of concern, especially in Africa, day market traders sometimes sell their products in the sun, making them easily exhausted, while their night counterparts are frequently exposed to mosquito bites, and possibly leading to malaria. Malaria control can only be successful and sustainable if the community regards the disease as very important, has accurate knowledge about it and the willingness to partake in its prevention and control [34]. Consequently, this study sought to determine the knowledge, attitudes, and practices on malaria in selected markets in Accra, Ghana, by exploring the perspectives of day and night traders of selected markets. To the best of our knowledge, this is the first report on malaria KAP with a focus on day and night market traders. This study provides important insights on the perspectives of day and night market traders on malaria, showing whether knowledge influences their choice of time to trade, and whether their time of trading makes a difference in their malaria related knowledge, attitudes, and practices.

## 2. Materials and Methods

### 2.1. Study Design, Area, and Population

A descriptive cross-sectional study was conducted among day and night traders in 10 selected markets in the Greater Accra Region, the region that hosts Accra, the capital and a very populous city of Ghana. The Greater Accra Region occupies a landmass of 3245 square kilometers, representing 1.4% of Ghana’s total landmass; according to the Ghana Statistical Service, it had a population of 4,943,075 that is unevenly distributed within the region’s 16 districts [35]. There are several markets in the region, with each district having at least one market. In this study, the markets in the region were stratified, and 10 markets were selected to give a better representation of traders who work in the markets within the region. The 10 markets chosen were major markets that congregate people from a variety of backgrounds and catchment areas coming to trade. This ensured that whatever findings on knowledge, attitudes and practices among the selected study population would be a true reflection of traders in these catchment areas.

The markets selected for the study were situated in Dodowa, Madina, Kwashieman, Kaneshie, Agbogbloshie, Makola, Korle-Bu, Tema Station, Tema Community 1, and roadside markets. At the various markets, respondents were selected using a systematic probability sampling method whereby every second consenting day and night market trader was interviewed.

Day traders usually start very early in the morning (around 5 a.m.) until early evening (around 5 p.m.), while night traders start from early evening until the next morning.

### 2.2. Sample Size

The minimum number of study participants was estimated by using the Cochran formula:

N = (Z2 × P (1 − P))/D2

where N = sample size, Z = standard statistic for a level of confidence of 95% (1.96), P = assumed true proportion of the population—50%, D = desired level of error margin—5% (0.05).

$$N\;=\;\frac{1.96^{2}\;\times \;0.50\;\left(1-0.50\right)}{0.05^{2}}$$

N = 384

The minimum sample size was therefore 384. However, 760 participants were sampled to further increase the power of the study.

### 2.3. Preparation of Questionnaire, Administration and Data Collection

Data were collected using a structured questionnaire with 40 questions grouped into 4 parts, comprising data on respondents’ socio-demographics and malaria-related knowledge, attitudes, and practices. Socio-demographic data were the first part of the questionnaire, and had 10 questions, which included age, gender, name of the market, marital status of respondent, frequency with which the respondent went to the market to trade each week, and products sold by the respondent. The second part, which determined the knowledge of respondents on malaria, had 14 items, including whether the respondent had heard of malaria, the respondent’s source of information on malaria, transmission routes and signs and symptoms of malaria known to the respondent, and groups vulnerable to malaria and preventive measures against the disease. The third part had 9 questions aimed to determine the attitude of the respondent toward malaria, for instance, the first thing the respondent would do if he or she experienced malaria-like symptoms, whether he or she always underwent laboratory tests to confirm his or her suspicion of having malaria, and the main factor that influenced his or her malaria-related health seeking behavior in response to experiencing malaria-like symptoms. The fourth part contained 7 questions, and sought to investigate the respondent’s practices on malaria, such as preferred type of malaria treatment (orthodox or herbal), choice of orthodox drugs for malaria treatment, use of insecticide-treated bed nets (ITN), and choice of malaria prevention measures (Supplementary Materials S1). The questionnaire was validated and pretested to allow for adjustments (such as correction of ambiguities and removal of irrelevancies) before the actual administration. Informed consent was also sought prior to questionnaire administration. The questionnaire was filled by the researcher in cases where the respondent was unable to write legibly. The language of communication was English or 2 commonly known local languages in Ghana (Twi and Ga languages), and it took a maximum of 25 min to fill each questionnaire, after which they were collected back immediately.

### 2.4. Data Analysis

Data collected were entered into Microsoft Office 2016 (Microsoft Corporation, Redmond, WA, USA), which was also used to generate all the charts. Characteristics of study respondents were described in the form of frequencies and percentages by the type of traders. Pearson’s chi-squared test was used to assess differences in distribution of respondents’ socio-demographic characteristics, individual knowledge items, overall knowledge levels, and attitudes of respondents towards malaria with regard to the types of traders. The test (Pearson’s chi-squared) was also used to assess the association between respondents’ socio-demographic characteristics and their overall knowledge levels, as well as between their attitudes and their malaria-related practices. All *p*-values less than 0.05 were considered statistically significant in the study. The respondents’ responses to the 12 knowledge questions were used to compute their composite knowledge level—each question had a score of 1 if the respondent answered correctly and a score of 0 if they responded wrongly. The scores were then categorized into 3 groups, with those with scores of 6 and below considered as having poor knowledge levels, those whose scores ranging from 7 to 9 considered to have moderate knowledge, and those who scored from 10 to 12 considered to have high knowledge on malaria (Supplementary Materials S2).

### 2.5. Ethical Considerations

Ethical approval was obtained from the Ethical and Protocol Review Committee of the College of Health Sciences, University of Ghana (Protocol Identification Number: CHS-Et/M.5–P 3.3/2016–2017). Informed consent was obtained from each respondent before filling out the questionnaires. All respondents were given assurance that the information they provided was purely intended for academic purposes. Codes were used for respondents rather than full names to ensure confidentiality and anonymity. Respondents were assured that the research would not involve any costs other than the time they would expend in responding to the questionnaire.

## 3. Results

### 3.1. Demographic Characteristics of Study Respondents

Out of the total of 760 study respondents interviewed, 253 (33.3%) were night traders and 507 (66.7%) were day traders. Of these, 65.79% (*n* = 500) were women and 28.4% (*n* = 216) individuals were within the age range of 26–30 years. Among the night traders, the highest and lowest proportions were recorded separately in the 26–30 years (24.5%, *n* = 62) and 36–40 years (6.3%, *n* = 16) age groups. A similar distribution for these age ranges was observed among the day traders (26–30 years (30.4%, *n* = 154); 36–40 years (4.5%, *n* = 45)) (Table 1).

Two hundred and eight respondents (representing 27.4%) were from the Madina market, whereas only a few (1.1%, *n* = 8) were from the Dodowa market. Among the night traders, 41.1% (*n* = 104) were from the Madina market, while no trader was from either of the Agbogbloshie, Tema Community 1, or Dodowa markets. Tema Community 1 recorded the highest proportion of day traders (32.4%, *n* = 164), whereas the Dodowa market recorded the lowest proportion (1.6%, *n* = 8). The distribution of night traders across the various markets was significantly different (χ^2^ = 181.2, *p* < 0.001) from that of the day traders (Table 1).

With regard to the number of times per week the traders went to the markets to trade, 8.9% (*n* = 68) went less than 4 times, 7.2% (*n* = 55) went 4 times, 41.6% (*n* = 316) went 5 times, 32.0% (*n* = 243) went 6 times, and 10.3% (*n* = 78) went 7 times; most of the night traders (39.1%, *n* = 99) went 6 times, and most of the day traders (44.4%, *n* = 225) went 5 times. Generally, there was a significant difference between the day and night market traders (χ^2^ = 12.43, *p* = 0.014) regarding the frequency with which they went to the markets to trade each week (Table 1). The 2 groups did not, however, differ significantly with reference to their distribution across gender (χ^2^ = 0.33, *p* = 0.564), marital status (χ^2^ = 2.60, *p* = 0.273), and products sold (χ^2^ = 3.97, *p* = 0.553) (Table 1).

### 3.2. Knowledge on Malaria as Exhibited by the Respondents

**Figure 1:**
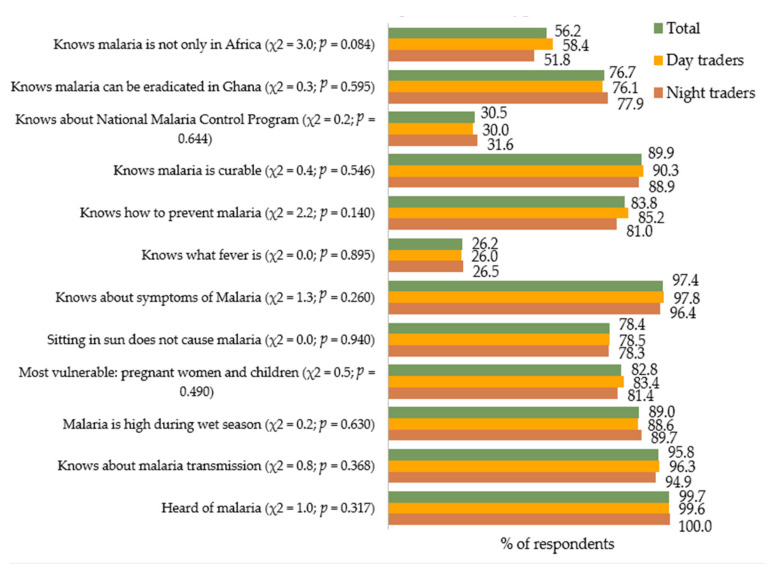
Proportions of respondents whose responses to the knowledge-assessing questions were accurate.

The proportion of respondents who answered each of the knowledge-assessing questions correctly is displayed in Figure 1. Almost all (99.7%) of the respondents had heard of malaria—100% of the night traders and 99.6% of the day traders.

With the study respondents’ sources of information on malaria, the day traders relative to the night traders, decreased across mass media platforms (radio, television, etc.) (54.4% vs. 55.3%), healthcare workers (21.1% vs. 21.0%), their homes (20.1% vs. 19.4%), other sources (2.8% each), and friends and neighbors (1.6% each). The difference in distribution between the 2 groups of traders was not significantly different (χ^2^ = 0.07, *p* = 0.999).

**Figure 2:**
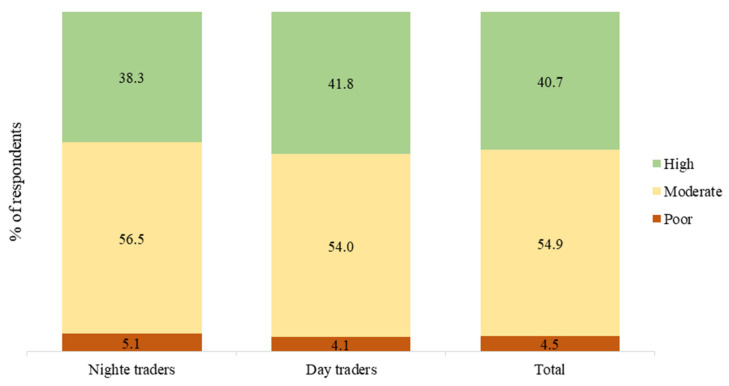
Level of knowledge on malaria among the study respondents.

In total, 95.8% of the study respondents (96.3% of day traders and 94.9% of night traders) knew of how malaria is transmitted. After computing a composite score for each study respondent regarding the 12 knowledge items shown in Figure 1, we found that 4.1% of the day traders had poor knowledge about malaria (scored 6 or below), whereas 54.0% and 38.3% respectively had moderate (scored 7 to 9) and high (scored 10 to 12) levels of knowledge on malaria (Figure 2). Among the night traders, poor knowledge was observed among 5.1% of the respondents, whereas 56.5% and 41.8% had moderate and high levels of knowledge on malaria, respectively. There was, however, no difference (χ^2^ = 1.06, *p* = 0.587) between the knowledge scores of the night traders and those of the day traders (Figure 2).

### 3.3. Associations Between Respondents’ Demographic Characteristics and Knowledge Levels

Demographic characteristics, such as traders’ gender (χ^2^ = 1.74, *p* = 0.420), marital status (χ^2^ = 3.57, *p* = 0.467), frequency of going to the market to trade (χ^2^ = 13.53, *p* = 0.095), and products sold (χ^2^ = 14.67, *p* = 0.145) were not significantly associated with their knowledge levels (Table 2). However, there was a significant association (χ^2^ = 20.97, *p* = 0.021) between the age groups of the traders and their levels of knowledge (Table 2). With regard to the traders’ age groups, the proportion of high knowledge level was largest among those in the range of 36–40 years (48.7%, 19/39), successively followed by those in the age group of 26–30 years (47.2%, 102/216), 31–35 years (39.8%, 70/176), above 40 years (38.2%, 29/76), 21–25 years (36.9%, 62/168), and 15–20 years (31.8%, 27/85).

When the number of traders with a high level of knowledge were stratified by the location of markets within which they plied their trade, we found that the largest proportion was recorded for the Dodowa market (75.0%, 6/8), followed by the Makola market (51.4%, 36/70); the lowest proportion was recorded for the roadside market (7.7%, 2/26). There was a significant association between the market location and the knowledge level of the study respondents (χ^2^ = 51.3, *p* < 0.001) (Table 2).

### 3.4. Attitudes of Respondents toward Malaria

It was observed that 9 out of every 10 respondents reported having ever experienced malaria (90.8%, *n* = 690). Among the two groups of traders, most (day traders = 212 (41.81%) and night traders = 102 (40.32%)) reported having had malaria at most, three times, whereas a few (day traders = 51 (10.06%) and night traders = 26 (10. 28%)) reported having had malaria for more than 10 times (Table 3).

The most common symptoms that informed the respondents’ suspicion of malaria were fever (day traders = 165 (32.54%) and night traders = 96 (37.94%)), body weakness (day traders = 134 (26.43%) and night traders = 60 (3.72%)), and loss of appetite (day traders = 122 (24.06%) and night traders = 57 (22.53%)) (Table 3). One-quarter (25.2%, *n* = 191) of the respondents indicated that they always took laboratory tests to confirm their malaria suspicion, whereas 46.8% (*n* = 335) proceeded to drug stores to obtain medications for malaria treatment solely on the basis of experiencing malaria-like symptoms (Table 3). The gravity of malaria-like symptoms was the predominantly reported motivating factor (79.8%, *n* = 565) for seeking malaria care. Majority of the respondents (95.0%, *n* = 707) reported having close friends who had ever had malaria, and only 4.4% (*n* = 32) had ever had malaria in the same period as their close friends (Table 3). None of the attitude factors showed a significant association (*p* > 0.05) with the two groups of market traders—night and day traders—investigated in this study (Table 3). In addition to the statistics presented in Table 2 and Table 3, multivariant analyses performed did not show significant difference between demographics and the respondents’ knowledge score (Supplementary Materials S3). 

### 3.5. Associations between Attitudes and Knowledge Levels of Respondents

Source of information on malaria (χ^2^ = 147.21, *p* < 0.01), use of laboratory tests to confirm malaria suspicion (χ^2^ = 11.66, *p* = 0.03), and the timing of malaria episodes between the study respondents and their friends (χ^2^ = 11.11, *p* = 0.004) were the factors associated with the respondents’ levels of knowledge on malaria (Table 4). Most of the traders with high knowledge of malaria obtained their malaria-related information from healthcare workers (49.38%, *n* = 79) and mass media platforms (radio, television, etc.) (43.51%, *n* = 181). Moreover, 43.98% (*n* = 250) of the study respondents who demonstrated high knowledge (43.98%, *n* = 250) did not always take laboratory tests to confirm their suspicion of malaria.

When the respondents were asked if their malaria episodes ever occurred at the same time as those of their friends, 41.80% (*n* = 293) of them, including those having high knowledge, answered “No”. The associations between the respondents’ responses to the attitude questions and their levels of knowledge are presented in Table 4.

### 3.6. Malaria-Related Practices, Including Treatment and Control Choices, among the Respondents

When the respondents were asked to choose between the use of orthodox or herbal remedies for malaria treatment, the majority of them (82.62% (*n* = 404) of the day traders and 83.79% (*n* = 212) of the night traders) indicated preference for orthodox medications. Moreover, most of them (75.66% (75.15% (*n* = 381) of the day traders and 76.68% (*n* = 197) of the night traders)) selected artemisinin-based combination therapy (ACT) as their preferred drug for malaria treatment among the range of orthodox medications presented to them (Table 5). Furthermore, use of insecticide-treated bed nets was reported by a minority (day traders = 185 (37.45%) and night traders = 107 (43.15%)) of the study respondents. The preferred malaria prevention method indicated by the majority of the respondents was insecticide spray/coil use (day traders = 274 (54.91%) and night traders = 134 (53.82%)). Most of the respondents (389/735) reported that the reason for their respective preferred malaria prevention measure was their perception of the choice as being of superior efficiency.

The majority of the respondents indicated a willingness to volunteer information about malaria to others (day traders = 302 (60.52%) and night traders = 136 (54.62%)). None of the malaria-related practice factors were, however, significantly associated with the type of traders (*p* > 0.05). Detailed information about malaria-related practices among the traders are outlined in Table 5.

### 3.7. Associations between Malaria-Related Practices and the Level of Knowledge

The associations between the respondents’ responses to the practice questions and their levels of knowledge are presented in Table 6. The best malaria treatment for respondents (χ^2^ = 26.5, *p* = 0.001), the preferred malaria treatment (χ^2^ = 10.7, *p* = 0.005), the use of insecticide-treated bed nets (χ^2^ = 9.8, *p* = 0.007), and the reasons for the preference of malaria prevention measures (χ^2^ = 28.7, *p* < 0.001) were the factors significantly associated with malaria knowledge (Table 6).

Most of the respondents who indicated orthodox medications as their preferred treatment for malaria had moderate knowledge about malaria (52.44%, *n* = 323), 43.51% (*n* = 268) had high knowledge, and 4.06% (*n* = 25) had poor knowledge (Table 6).

Similarly, most of those who indicated ACTs as their preferred choice for malaria treatment had moderate knowledge about malaria (50.61%, *n* = 291), 45.22% (*n* = 260) had high knowledge, and 4.17% (*n* = 24) had poor knowledge (Table 6).

In much the same way, most of those who indicated that they do not use insecticide-treated bed nets had moderate knowledge about malaria (57.33%, *n* = 258), 37.11% (*n* = 167) had high knowledge, and 5.56% (*n* = 25) had poor knowledge (Table 6).

Furthermore, most of those who revealed their reason for preferred malaria prevention measure to be their perception of the choice as being of superior efficiency had moderate knowledge about malaria (52.44%, *n* = 204), 44.73% (*n* = 174) had high knowledge, and 2.83% (*n* = 11) had poor knowledge (Table 6).

## 4. Discussion

Malaria is of immense public health importance, particularly, in malaria-endemic regions, such as sub-Saharan Africa, where it causes significant morbidity and mortality amongst vulnerable groups [4]. The success of efforts aimed at reducing the burden of the disease could be sustained and improved if inhabitants of these regions are well-informed about the disease, including its route of transmission, signs and symptoms, management, and preventive measures [34]. In line with this, this study sought to unravel the perspectives of an important, but largely overlooked, section of the Ghanaian population—day and night market traders—with regards to their malaria-related knowledge, attitudes, and practices. This is the first malaria KAP report focused on the perspectives of day and night market traders in the country, and respondents were recruited from selected markets within the Greater Accra Region.

The study’s recording of more respondents among day traders compared to night traders, in a ratio of about 2:1, is an observation that depicts the typical nature of Ghanaian markets, which are mostly active in the day, with a large amount of people going there for business. Hence, it is not surprising that a greater proportion of the respondents were day traders. Moreover, most of the study respondents were from the Madina market, whereas a few were from the Dodowa market, and this reflects the geographical distribution of the towns hosting these two markets—unlike Madina, which is closer to central Accra, Dodowa is at the periphery of the region, and seems to have fewer inhabitants.

With reference to knowledge in this study, the observation that almost all (99.7%) of the respondents had heard about malaria is very encouraging, and is consistent with the finding of Singh et al. [29], in whose study the majority (93.5%) of the respondents had heard of malaria. Similar findings have been reported by Amusan et al. [36] and Munisi et al. [37], who respectively indicated that 93.9% and 97.7% of the respondents they interviewed had heard of malaria. The homogeneity of this observation among the current study and those of Singh et al. [29], Amusan et al. [36], and Munisi et al. [37] is consistent with the endemicity of the disease in sub-Saharan Africa.

Still on the topic of knowledge, when respondents were asked about the transmission vector, a high knowledge (95.8%) on the vector was observed, indicating their appreciation of the connection between the disease and bite of mosquitoes. This high level of knowledge on the vector is similar to the 96.9% reported by Amusan et al. [36] and 95.31% by Munisi et al. [37]. On the contrary, a study by Okwa et al. [18], which assessed the KAP of malaria among 50 artisans and 50 traders in selected areas in Lagos, Nigeria, observed that only 4% (*n* = 2) of the artisans and 52% (*n* = 26) of the traders studied made a connection between malaria and mosquitoes. This disparity in knowledge on transmission vectors was attributed to a reflection of the low educational level of Okwa et al.’s [18] study population. Knowing the vector responsible for malaria transmission is critical in the control of the disease, and hence is a welcomed observation of the current study.

An interesting perspective was unraveled when respondents were asked if sitting in the sun for long hours caused malaria. Similar proportions of the day (21.5%) and night (21.7%) market traders answered in the affirmative, indicating that misconceptions about the sun and malaria might exist among the market traders. They reasoned that since fever (high body temperature) is associated with malaria, sitting on the sun for a long time would automatically lead to malaria. However, this misconception was held by similar proportions of day and night market traders (21.5% of the day traders and 21.7% of the night traders), suggesting that it had little or no influence on the traders’ choice of time to be at the markets.

Another intriguing perception uncovered during the interactions with the participants was that the word “fever” seems to be synonymously used to mean “malaria”. It is therefore “normal” for someone who wants to say “I have got malaria” to say “I have got fever”. Moreover, being in malaria-endemic communities, where most individuals would have had prior exposure to malaria by the time they reach trading age, it appears as though the respondents do not regard the disease as an extremely dangerous condition. Therefore, it is not surprising for an individual who says “I just had a little fever” to actually mean “I simply had malaria, and nothing more serious than that”.

Therefore, education needs to be directed at these misconceptions to improve overall knowledge about malaria. In contrast, in Godwin et al.’s [7] study, which was conducted in a malaria-endemic city of North Central Nigeria, only a few of the respondents (less than 0.1%) had such a misconception. In their report, the authors attributed the high knowledge among the study respondents to marriage, educational level, and some occupations of the respondents (being teachers and health workers). In another study conducted in Nigeria among market women, the proportion of respondents who had the noted “malaria–sun” misconception was 12.2% [8].

Knowledge about signs and symptoms of malaria among the respondents in the current study was nonetheless very good, with 96.4% of the night market traders and 97.8% of day traders knowing about malaria symptoms, which is consistent with the findings of Singh et al. [29] in Nigeria, Mbohou et al. [17] in Cameroon, and Owusu et al. [23] in Ghana. Having sound knowledge about signs and symptoms of the disease is helpful to its diagnosis, treatment, and management.

After computing the overall knowledge score in this study, more than half of the traders had moderate knowledge on malaria, with 54.9% and 40.7% of the respondents respectively having moderate and high levels of knowledge on malaria. This is consistent with findings reported by Ismail et al. [38] among the majority of primary healthcare workers in Plateau State, Nigeria. Adegun et al. [39] also reported an overall moderate to a high level of knowledge on malaria, malaria-related issues, and malaria preventive practices among migrant farmers. However, a high overall knowledge was reported among primary school children in Bagamoya, Tanzania [27]; among employees from enterprises in Douala, Cameroon [17]; among malaria symptomatic patients in a referral hospital in Tanzania [37]; and prescribers in Ghana [22]. This suggests that there is still room for improvement on the knowledge of day and night market traders in the Greater Accra Region and other groups of people sampled in the cited studies. On the contrary, a low overall knowledge was reported by Okwa et al. [18] among artisans and traders and Owusu et al. [23] among people living with HIV in rural communities in Ghana. These findings were attributed to the level of education of the artisans and traders and the rural living area of the respondents, respectively. Moreover, the difference between the knowledge level observed in the current study and that of Owusu et al. [23], which was also conducted in Ghana, may be because the respondents in Owusu et al.’s [23] study were inhabiting a rural area, unlike the current study whose respondents were sampled predominantly from urban areas.

Identifying mass media platforms and health workers as the means by which the majority of the respondents acquired their overall knowledge is not surprising, because in the capital city there is easy access to information; in fact, almost all the traders had heard about malaria. These traders usually commute to and from the markets via public transport systems, from whence they are often exposed to radio- and television-hosted talkshows, some of which are centered on malaria. The 2019 Ghana Malaria Indicator Survey reported that the majority (82%) of the respondents revealed that they had been presented with messages on malaria on television or radio. Besides the aforementioned avenues for accessing malaria-related information, loud public address systems, which are common features of marketplaces in the country, form part of the mass media platforms through which more than half of the market traders of the current study (54.4%) obtained their malaria-related information. A similar explanation regarding mass media platforms was proffered by Sumari et al. [27], who attributed the high level of malaria knowledge among the school children in their study to national and local public awareness programs through mass media platforms like radio and television.

Similar to findings reported by Sumari et al. [27], this study showed that the sources of information on malaria were associated with the level of knowledge on malaria, with mass media platforms and health workers being the most common sources of information reported by the respondents. This study’s finding indicates that respondents’ source of information might determine whether they would have low, moderate, or high knowledge of malaria. Meanwhile, there was no observed difference between day and night market traders’ sources of information, possibly explaining why there was no difference in the knowledge level between the two groups. However, this is a good observation, since it might imply that no matter the time of trading at the market or being at home, there is the chance of receiving some information on malaria.

When the associations between the demographic characteristics of the day and night market traders and their knowledge level were explored, age group and specific markets the respondents traded at were found to be significantly associated with their knowledge level. Regarding the age groups, most of the respondents aged 26 years and above had a high knowledge, with a large proportion of these being in the 36–40 years age group. This is in contrast to a study by Jimam and Ismail [40], who determined the predictive factors of KAP on uncomplicated malaria. Although those researchers showed that age was significantly associated with the level of knowledge, as was seen in this study, they revealed a likely decrease in KAP with increased age of the patients. This disparity could be as a result differences in study population and setting. The high knowledge level on malaria among those trading at the Dodowa, Makola, Tema Community 1, and Madina markets in comparison with the roadside market, which had the least proportion of traders with high knowledge could be as a result of the location of the market. Dodowa, Makola, Tema Community 1, and Madina are relatively more cosmopolitan than the roadside market. Moreover, as indicated earlier, at the market, there is usually loud radio being played, which may carry information on malaria awareness, and those there may have access to such information unlike those at the roadside.

With regards to respondents’ attitudes toward malaria in this study, 9 out of every 10 respondents reported having ever experienced malaria, further buttressing the view that malaria is endemic in this region [4] and that education and interventions need to be strengthened to curb this disease. A significant proportion (67.46%) indicated going to the drug store/chemical shop (46.79%, *n* = 335) and practicing self-treatment at home (15.92%, *n* = 114) when they experience malaria-like symptoms, similar to what was observed by Adedotun et al. [41]. They reported that in their study that about 90% of suspected malaria cases in children and adults were first treated at home with herbal medications or drugs purchased from drug stores/chemical shops [41]. Similarly, Okwa et al. [18] and Munisi et al. [37] recorded that most respondents in their study indicated self-medication and buying drugs from drug stores, respectively, when asked their initial action they took in response to malaria-like symptoms. On the contrary, studies by Mbohou et al. [17] and Sumari et al. [27] reported that the majority of their respondents indicated going to the hospital as their first response. However, in this study, only (233, 32.54%) indicated going to the hospital as their first response. According to the WHO [42], early diagnosis and prompt treatment of malaria are crucial to prevent severe disease and death hence the first response to malaria symptoms is important. In this study, the initial response of going to a drug store, self-medication, and doing nothing about the malaria-like symptoms was indicated by a large proportion (67.46%) of the study respondents, revealing a clear deficiency in attitude. Therefore, the market traders in the region need to be educated on appropriate measures to take when experiencing malaria-like symptoms, as recommended by the WHO [37].

When the traders were asked about the important factors that prompted their seeking malaria care at the hospital, the gravity of symptoms was the most cited factor (79.8%), and this is similar to what Singh et al. [29] reported. In this study, none of the respondents’ attitudes showed a significant association with the two types of traders. This similarity in attitude could be as a result of both groups having no difference in their knowledge level as observed in this study. Furthermore, associating the level of knowledge on malaria to the attitude of traders revealed that the use of laboratory tests to confirm malaria status (*p* = 0.003) was significantly associated with the level of knowledge on malaria, an observation of great importance. However, it was observed that 43.94% of traders with high knowledge of malaria did not always take a laboratory test to confirm malaria. Thus, it can be inferred that having a high knowledge of malaria does not translate into a positive attitude. This finding is of great concern, and therefore behavior change communication strategies may need to be used to ensure taking a laboratory test to confirm malaria before commencing treatment. It is recommended by WHO that all individuals with signs and symptoms suggestive of malaria should have a confirmatory laboratory test, either via microscopy or rapid diagnostic test [37].

With regard to the respondents’ practices on malaria, most of them reported using at least one malaria preventive method, with the majority indicating what seems to be the most common in the Greater Accra Region as their preferred malaria preventive methods—insecticide spray/coil (408, 54.55%) and use of bed nets (232, 31.02%). These two preventive methods were similarly reported by Mbohou et al. [17], Singh et al. [29], and Munisi et al. [37] as the major methods used among their study respondents. Insecticide spray/coil are, however, the most used preventive method in this study, and similar findings were reported by Okwa et al. [18] who explained that this preference in their study population was due to the higher availability and affordability of mosquito coils and insecticide sprays. In this study, the reasons why traders chose their respective preferred malaria preventive methods were significantly associated with their knowledge level and the main reasons indicated were the measure being more effective and easier to access.

The use of treated bed nets was significantly associated with the knowledge level of traders. However, out of 450 traders (60.65%) who do not use treated bed nets, 167 (37.11%) had an overall high knowledge level, proving to be a worrying observation. The most common reason given for not using treated bed nets was that it was associated with discomfort, and this was consistent with findings reported by Owusu et al. [23], who revealed that fewer people used insecticide-treated nets among their study population with the reason being that it was “too hot” to sleep under. Meanwhile, in the current study, all traders indicated using at least one malaria preventive method. However, education on the importance of consistent use of at least one malaria preventive method is crucial to reduce the transmission of malaria. In this study, none of the reported practices showed a significant association between the two groups of traders, an observation that could be as a result of both groups having no difference in their knowledge and attitude towards malaria.

Furthermore, with associations between the level of knowledge on malaria and practices of traders, it was revealed that the traders’ choice of drug for malaria treatment (*p* = 0.001) and preferred malaria treatment type (orthodox or herbal) (*p* = 0.005) were significantly associated with their knowledge level. The majority of the respondents (575, 75.65%) indicated ACTs as their preferred choice of malaria treatment drug, with 45.22% of this group having a high overall knowledge level and 50.61% having a moderate overall knowledge level on malaria, suggestive of a positive translation of knowledge to practice. Moreover, when asked of preferred treatment type, the majority (83%) of the respondents indicated orthodox, while 17% indicated herbal medication, an observation consistent with Mbohou et al. [17], who revealed that only a small fraction (8.6%) of respondents in their study were resorting to traditional medicine (herbs and parts of plants), and this was attributed to socio-cultural beliefs and practices. On the contrary, Okwa et al. [18] revealed that the majority of traders in their study used local herbs. The disparity in findings between this study and the findings of Okwa et al. [18] could be because their study reported an overall low knowledge level among their study respondents, while this study revealed an overall moderate and high level of knowledge among the day and night market workers.

## 5. Conclusions

This study showed no significant difference between the perspectives of the day and night market traders in terms of knowledge, attitudes, and practices towards malaria. The study concludes that day and night market traders have similar knowledge levels, with the majority of them having moderate knowledge of malaria. However, misconceptions about malaria (especially as caused by exposure to the sun) still exist among the market traders in the Greater Accra Region, which seems to be influencing their attitudes and practices. When asked their initial reaction to malaria-like symptoms, many indicated going to a drug store, self-medication, and doing nothing. This suggests that the overall moderate knowledge did not translate into a positive attitude. Moreover, the majority of the market traders with high knowledge levels did not always take a laboratory test for confirmation of malaria, which was not the best of practices. Therefore, there is a need for appropriate health education programs and interventions directed at misconceptions, poor attitudes, and poor practices revealed by this study. Moreover, it would be appropriate for traders to be educated on measures such as use of topical medications that would help prevent bite from mosquitoes whilst trading at the market, especially during the night. This may lead to behavioral changes and ultimately help in the prevention and control of malaria in Ghana, and globally.

Limitations of the study:

This study relied on self-reportage, therefore the veracity of the information given cannot be guaranteed. Since this is a cross-sectional study, which took a snapshot of the population at a specific time, the proper representation of the study population cannot be totally guaranteed. Cross-sectional studies also do not analyze the behavior over a period of time. Therefore, their attitude and practice cannot be guaranteed in absolute terms. Knowledge level could not be attributed to educational level in this study because educational level was not a target in the questionnaire developed. Moreover, the inability to calculate the Cronbach’s alpha can be considered a limitation of the study. Finally, the questionnaire did not explore the participant’s perception on the seriousness of the disease.

Strengths of the study:

In addition to this study being the first to focus on day and night market traders, high power of the study (large sample size) can be considered a strength of the study.

## Figures and Tables

**Table 1:** Demographic characteristics of the study respondents.

Characteristics	Night Traders	Day Traders	Total	χ^2^	*p*-Value
	*n* (%)	*n* (%)	*n* (%)		
**Age Group**				8.72	0.121
15–20 years	36 (14.23)	49 (9.66)	85 (11.18)		
21–25 years	53 (20.95)	115 (22.68)	168 (22.11)		
26–30 years	62 (24.51)	154 (30.37)	216 (28.42)		
31–35 years	55 (21.74)	121 (23.87)	176 (23.16)		
36–40 years	16 (6.32)	23 (4.54)	39 (5.13)		
>40 years	31 (12.25)	45 (8.88)	76 (10.00)		
**Gender**				0.33	0.564
Male	83 (32.81)	177 (34.91)	260 (34.21)		
Female	170 (67.19)	330 (65.09)	500 (65.79)		
**Marital Status**				2.60	0.273
Married	100 (39.53)	200 (39.45)	300 (39.47)		
Never married/single	139 (54.94)	291 (57.40)	430 (56.58)		
Widowed/divorced/separated	14 (5.53)	16 (3.16)	30 (3.95)		
**Market**				181.22	<0.001 ***
Agbogbloshie	0 (0.00)	56 (11.05)	56 (7.37)		
Tema Community 1	0 (0.00)	164 (32.35)	164 (21.58)		
Dodowa	0 (0.00)	8 (1.58)	8 (1.05)		
Kaneshie	5 (1.98)	30 (5.92)	35 (4.61)		
Korle-Bu	30 (11.86)	30 (5.92)	60 (7.89)		
Kwashieman	27 (10.67)	27 (5.33)	54 (7.11)		
Madina	104 (41.11)	104 (20.51)	208 (27.37)		
Makola	35 (13.83)	35 (6.90)	70 (9.21)		
Roadside market	13 (5.14)	13 (2.56)	26 (3.42)		
Tema Station	39 (15.42)	40 (7.89)	79 (10.39)		
**Products Sold**				3.97	0.553
Prepared food	53 (20.95)	97 (19.13)	150 (19.74)		
Fruits and vegetables	44 (17.39)	103 (20.32)	147 (19.34)		
Clothes	20 (7.91)	56 (11.05)	76 (10.00)		
Drinks	40 (15.81)	72 (14.20)	112 (14.74)		
Electricals and electronics	21 (8.30)	47 (9.27)	68 (8.95)		
Other	75 (29.64)	132 (26.04)	207 (27.24)		
**Frequency with which Traders went to the Market to Trade Each Week**				12.43	0.014 *
<4 times	17 (6.72)	51 (10.06)	68 (8.95)		
4 times	16 (6.32)	39 (7.69)	55 (7.24)		
5 times	91 (35.97)	225 (44.38)	316 (41.58)		
6 times	99 (39.13)	144 (28.40)	243 (31.97)		
7 times	30 (11.86)	48 (9.47)	78 (10.26)		

*n*: frequency, %: percentage, χ^2^: chi-squared, *: *p* value < 0.05, ***: *p* value < 0.01.

**Table 2:** Associations between demographic characteristics and knowledge levels of respondents.

		Level of Knowledge on Malaria				
Characteristics	Total	Poor	Moderate	High	χ^2^	*p*-Value
	*n*	*n* (%)	*n* (%)	*n* (%)		
**Age Group**					20.97	0.021 *
15–20 years	85	2 (2.35)	56 (65.88)	27 (31.76)		
21–25 years	168	13 (7.74)	93 (55.36)	62 (36.90)		
26–30 years	216	3 (1.39)	111 (51.39)	102 (47.22)		
31–35 years	176	11 (6.25)	95 (53.98)	70 (39.77)		
36–40 years	39	0 (0.00)	20 (51.28)	19 (48.72)		
>40 years	76	5 (6.58)	42 (55.26)	29 (38.16)		
**Gender**					1.74	0.420
Male	260	10 (3.85)	151 (58.08)	99 (38.08)		
Female	500	24 (4.80)	266 (53.20)	210 (42.00)		
**Marital Status**					3.57	0.467
Married	300	10 (3.33)	166 (55.33)	124 (41.33)		
Never married/single	430	24 (5.58)	234 (54.42)	172 (40.00)		
Widowed/divorced/separated	30	0 (0.00)	17 (56.67)	13 (43.33)		
**Market**					51.27	<0.001 ***
Agbogbloshie	56	1 (1.79)	31 (55.36)	24 (42.86)		
Tema Community 1	164	7 (4.27)	81 (49.39)	76 (46.34)		
Dodowa	8	0 (0.00)	2 (25.00)	6 (75.00)		
Kaneshie	35	0 (0.00)	26 (74.29)	9 (25.71)		
Korle-Bu	60	0 (0.00)	34 (56.67)	26 (43.33)		
Kwashieman	54	4 (7.41)	26 (48.15)	24 (44.44)		
Madina	208	16 (7.69)	124 (59.62)	68 (32.69)		
Makola	70	0 (0.00)	34 (48.57)	36 (51.43)		
Roadside market	26	0 (0.00)	24 (92.31)	2 (7.69)		
Tema Station	79	6 (7.59)	35 (44.30)	38 (48.10)		
**Products Sold by the Traders**					14.67	0.145
Prepared food	150	4 (2.67)	76 (50.67)	70 (46.67)		
Fruits and vegetables	147	8 (5.44)	83 (56.46)	56 (38.10)		
Clothes	76	1 (1.32)	44 (57.89)	31 (40.79)		
Drinks	112	2 (1.79)	64 (57.14)	46 (41.07)		
Electricals and electronics	68	6 (8.82)	42 (61.76)	20 (29.41)		
Other	207	13 (6.28)	108 (52.17)	86 (41.55)		
**Number of Times Respondents Went to the Market to Trade**					13.53	0.095
<4 times	68	0 (0.00)	38 (55.88)	30 (44.12)		
4 times	55	2 (3.64)	34 (61.82)	19 (34.55)		
5 times	316	21 (6.65)	174 (55.06)	121 (38.29)		
6 times	243	11 (4.53)	124 (51.03)	108 (44.44)		
7 times	78	0 (0.00)	47 (60.26)	31 (39.74)		

*n*: frequency, %: percentage, χ^2^: chi-squared, *: *p* value < 0.05, ***: *p* value < 0.01.

**Table 3:** Attitudes of the study respondents toward malaria.

Characteristics	Night Traders	Day Traders	Total	χ^2^	*p*-Value
	*n* (%)	*n* (%)	*n* (%)		
**Respondents’ Affirmation of Malaria History**				0.03	0.853
Yes	229 (90.51)	461 (90.93)	690 (90.79)		
No	24 (9.49)	46 (9.07)	70 (9.21)		
**Number of Times Respondents had had Malaria**				0.56	0.906
1–3 times	102 (40.32)	212 (41.81)	314 (41.32)		
4–6 times	95 (37.55)	178 (35.11)	273 (35.92)		
7–10 times	30 (11.86)	66 (13.02)	96 (12.63)		
More than 10 times	26 (10.28)	51 (10.06)	77 (10.13)		
**Symptoms that Informed Respondents’ Malaria Suspicion**				3.85	0.698
Fever	96 (37.94)	165 (32.54)	261 (34.34)		
Vomiting	17 (6.72)	44 (8.68)	61 (8.03)		
Weakness	60 (23.72)	134 (26.43)	194 (25.53)		
Loss of appetite	57 (22.53)	122 (24.06)	179 (23.55)		
Anemia	14 (5.53)	23 (4.54)	37 (4.87)		
No idea	7 (2.77)	12 (2.37)	19 (2.50)		
Others	2 (0.79)	7 (1.38)	9 (1.18)		
**Respondents’ Reliance on Laboratory Tests for Confirmation of Malaria Suspicion**				0.08	0.779
Yes	62 (24.51)	129 (25.44)	191 (25.13)		
No	191 (75.49)	378 (74.56)	569 (74.87)		
**The First Thing Respondents Did Upon Experiencing Malaria-Like Symptoms**				1.37	0.712
Visiting the hospital	76 (31.93)	157 (32.85)	233 (32.54)		
Visiting the drug store	113 (47.48)	222 (46.44)	335 (46.79)		
Self-medication at home	35 (14.71)	79 (16.53)	114 (15.92)		
Disregard symptoms	14 (5.88)	20 (4.18)	34 (4.75)		
**Factors that Promoted Respondents’ Malaria Care Seeking**				0.73	0.865
Gravity of symptoms	187 (79.24)	378 (80.08)	565 (79.80)		
Cost of hospital care	6 (2.54)	14 (2.97)	20 (2.82)		
Duration of symptoms	29 (12.29)	49 (10.38)	78 (11.02)		
Availability of time	14 (5.93)	31 (6.57)	45 (6.36)		
**Respondents’ Responses Regarding Having Close Friends Who had had Malaria**				0.26	0.609
Yes	239 (95.60)	468 (94.74)	707 (95.03)		
No	11 (4.40)	26 (5.26)	37 (4.97)		
**Respondents’ Malaria Episodes Sometimes Coincided with their Friends’ Malaria Episodes**				0.16	0.691
Yes	10 (3.95)	22 (4.58)	32 (4.37)		
No	243 (96.05)	458 (95.42)	701 (95.63)		

*n*: frequency, %: percentage, χ^2^: chi-squared.

**Table 4:** Associations between respondents’ attitudes toward malaria and their knowledge levels.

		Level of Knowledge on Malaria			χ^2^	*p*-Value
Characteristics	Total	Poor	Moderate	High		
	*N*	*n* (%)	*n* (%)	*n* (%)		
**Sources of Information on Malaria**					147.21	<0.001 *
Home	151	5 (3.31)	98 (64.90)	48 (31.79)		
Family and neighbors	12	7 (58.33)	5 (41.67)	0 (0.00)		
Health workers	160	7 (4.38)	74 (46.25)	79 (49.38)		
Mass media (radio, TV, etc.)	416	8 (1.92)	227 (54.57)	181 (43.51)		
Other	21	7 (33.33)	13 (61.90)	1 (4.76)		
**Respondents’ Affirmation of Malaria History**					1.38	0.501
Yes	690	31 (4.49)	374 (54.20)	285 (41.30)		
No	70	3 (4.29)	43 (61.43)	24 (34.29)		
**Number of Times Respondents had had Malaria**					10.30	0.112
1–3 times	314	13 (4.14)	155 (49.36)	146 (46.50)		
4–6 times	273	13 (4.76)	164 (60.07)	96 (35.16)		
7–10 times	96	3 (3.13)	52 (54.17)	41 (42.71)		
More than 10 times	77	5 (6.49)	46 (59.74)	26 (33.77)		
**Symptoms that Informed Respondents’ Malaria Suspicion**					14.12	0.293
Fever	261	12 (4.60)	141 (54.02)	108 (41.38)		
Vomiting	61	2 (3.28)	41 (67.21)	18 (29.51)		
Weakness	194	11 (5.67)	100 (51.55)	83 (42.78)		
Loss of appetite	179	6 (3.35)	95 (53.07)	78 (43.58)		
Anemia	37	3 (8.11)	18 (48.65)	16 (43.24)		
No idea	19	0 (0.00)	15 (78.95)	4 (21.05)		
Others	9	0 (0.00)	7 (77.78)	2 (22.22)		
**Respondents’ Reliance on Laboratory Tests for Confirmation of Malaria Suspicion**					11.66	0.003 *
Yes	191	13 (6.81)	119 (62.30)	59 (30.89)		
No	569	21 (3.69)	298 (52.37)	250 (43.94)		
**The First Thing Respondents Did Upon Experiencing Malaria-Like Symptoms**					5.97	0.427
Visiting the hospital	233	11 (4.72)	119 (51.07)	103 (44.21)		
Visiting the drug store	335	15 (4.48)	177 (52.84)	143 (42.69)		
Self-medication at home	114	5 (4.39)	71 (62.28)	38 (33.33)		
Disregard symptoms	34	0 (0.00)	20 (58.82)	14 (41.18)		
**Factors that Promoted Respondents’ Malaria Care Seeking**					5.12	0.528
Gravity of symptoms	565	28 (4.96)	302 (53.45)	235 (41.59)		
Cost of hospital care	20	0 (0.00)	13 (65.00)	7 (35.00)		
Duration of symptoms	78	3 (3.85)	47 (60.26)	28 (35.90)		
Availability of time	45	0 (0.00)	26 (57.78)	19 (42.22)		
**Respondents’ Responses Regarding Having Close Friends Who had had Malaria**					5.19	0.075
Yes	707	29 (4.10)	381 (53.89)	297 (42.01)		
No	37	3 (8.11)	25 (67.57)	9 (24.32)		
**Respondents’ Malaria Episodes Sometimes Coincided with their Friends’ Malaria Episodes**					11.11	0.004 *
Yes	32	5 (15.63)	17 (53.13)	10 (31.25)		
No	701	26 (3.71)	382 (54.49)	293 (41.80)		

TV: television, *n*: frequency, %: percentage, χ^2^: chi-squared, *: *p* value < 0.05.

**Table 5:** Practices of respondents regarding malaria.

	Night Traders	Day Traders	Total	χ^2^	*p*-Value
Characteristics	*n* (%)	*n* (%)	*n* (%)		
**Respondents’ Choice of Drugs for Malaria Treatment**				6.13	0.189
ACTs	194 (76.68)	381 (75.15)	575 (75.66)		
Chloroquine	10 (3.95)	23 (4.54)	33 (4.34)		
Paracetamol	12 (4.74)	20 (3.94)	32 (4.21)		
Herbal	26 (10.28)	39 (7.69)	65 (8.55)		
Other	11 (4.35)	44 (8.68)	55 (7.24)		
**Respondents’ Preferred Type of Malaria Treatment**				0.16	0.686
Orthodox	212 (83.79)	404 (82.62)	616 (83.02)		
Herbal	41 (16.21)	85 (17.38)	126 (16.98)		
**Use of Insecticide-Treated Bed Nets**				2.24	0.134
Yes	107 (43.15)	185 (37.45)	292 (39.35)		
No	141 (56.85)	309 (62.55)	450 (60.65)		
**Rationale for Non-Use of Treated Bed Nets**				3.01	0.556
Discomfort	99 (66.89)	200 (62.50)	299 (63.89)		
Allergy to content of the nets	30 (20.27)	59 (18.44)	89 (19.02)		
Perception of bed nets as sources of illness	1 (0.68)	3 (0.94)	4 (0.85)		
No particular reason	15 (10.14)	45 (14.06)	60 (12.82)		
Other	3 (2.03)	13 (4.06)	16 (3.42)		
**Preferred Malaria Prevention Measures**				3.71	0.447
Clearing of bushes and stagnant water	19 (7.63)	54 (10.82)	73 (9.76)		
Residual spraying	10 (4.02)	19 (3.81)	29 (3.88)		
Use of insecticide-treated bed nets	85 (34.14)	147 (29.46)	232 (31.02)		
Insecticide spray/coil	134 (53.82)	274 (54.91)	408 (54.55)		
Other	1 (0.40)	5 (1.00)	6 (0.80)		
**Reason for Chosen Malaria Prevention Measure**				3.09	0.543
Perceived lack of side effects	11 (4.49)	22 (4.49)	33 (4.49)		
Perceived superior efficiency	132 (53.88)	257 (52.45)	389 (52.93)		
Lower cost of choice	19 (7.76)	58 (11.84)	77 (10.48)		
Easy access to choice	61 (24.90)	110 (22.45)	171 (23.27)		
Owing to recommendations by others	22 (8.98)	43 (8.78)	65 (8.84)		
**Willingness to Volunteer Information about Malaria to Others**				2.38	0.123
Yes	136 (54.62)	302 (60.52)	438 (58.56)		
No	113 (45.38)	197 (39.48)	310 (41.44)		

*n*: frequency, %: percentage, χ^2^: chi-squared, ACT: artemisinin-based combination therapy.

**Table 6:** Associations between respondents’ practices and levels of knowledge on malaria.

		Level of Knowledge about Malaria				
Characteristics	Total	Poor	Moderate	High	χ^2^	*p*-Value
	*n*	*n* (%)	*n* (%)	*n* (%)		
**Respondents’ Choice of Drugs for Malaria Treatment**					26.49	0.001 *
ACTs	575	24 (4.17)	291 (50.61)	260 (45.22)		
Chloroquine	33	1 (3.03)	25 (75.76)	7 (21.21)		
Paracetamol	32	0 (0.00)	20 (62.50)	12 (37.50)		
Herbal	55	5 (9.09)	36 (65.45)	14 (25.45)		
Other	65	4 (6.15)	45 (69.23)	16 (24.62)		
**Respondents’ Preferred Type of Malaria Treatment**					10.74	0.005 *
Orthodox	616	25 (4.06)	323 (52.44)	268 (43.51)		
Herbal	126	7 (5.56)	84 (66.67)	35 (27.78)		
**Use of Insecticide-Treated Bed Nets**					9.80	0.007 *
Yes	292	8 (2.74)	145 (49.66)	139 (47.60)		
No	450	25 (5.56)	258 (57.33)	167 (37.11)		
**Rationale for Non-Use of Treated Bed Nets**					4.65	0.794
Discomfort	299	14 (4.68)	165 (55.18)	120 (40.13)		
Allergy to content of the nets	89	6 (6.74)	52 (58.43)	31 (34.83)		
Perception of bed nets as sources of illness	4	0 (0.00)	2 (50.00)	2 (50.00)		
No particular reason	60	2 (3.33)	35 (58.33)	23 (38.33)		
Other	16	1 (6.25)	12 (75.00)	3 (18.75)		
**Preferred Malaria Prevention Measures**					13.06	0.110
Clearing of bushes and stagnant water	73	5 (6.85)	40 (54.79)	28 (38.36)		
Residual spraying	29	3 (10.34)	17 (58.62)	9 (31.03)		
Use of insecticide-treated bed nets	232	6 (2.59)	114 (49.14)	112 (48.28)		
Insecticide spray/coil	408	18 (4.41)	231 (56.62)	159 (38.97)		
Other	6	0 (0.00)	5 (83.33)	1 (16.67)		
**Reason for Chosen Malaria Prevention Measure**					28.65	<0.001 *
Perceived lack of side effects	33	0 (0.00)	17 (51.52)	16 (48.48)		
Perceived superior efficiency	389	11 (2.83)	204 (52.44)	174 (44.73)		
Lower cost of choice	77	1 (1.30)	52 (67.53)	24 (31.17)		
Easy access to choice	171	17 (9.94)	86 (50.29)	68 (39.77)		
Owing to recommendations by others	65	1 (1.54)	42 (64.62)	22 (33.85)		
**Willingness to Volunteer Information about Malaria to Others**					3.26	0.196
Yes	438	23 (5.25)	230 (52.51)	185 (42.24)		
No	310	9 (2.90)	177 (57.10)	124 (40.00)		

*n*: frequency, %: percentage, χ^2^: chi-squared, *: *p* value < 0.05.

## Data Availability

The data presented in this study are available on request from the corresponding authors; patborket2002@yahoo.com or pbtetteh-quarcoo@ug.edu.gh (P.B.T.-Q.); ntkddayie@ug.edu.gh (N.T.K.D.D.); Tel.: +233-244633251 (P.B.T.-Q.); +233-208449415 (N.T.K.D.D.).

## Supplementary Materials

The following are available online at https://www.mdpi.com/article/10.3390/ijerph18073468/s1, Table S1: Questionnaire: Knowledge, attitude, practices (Kap) on malaria and plasmodium parasite carriage among day and night market workers in Greater Accra, Ghana: A cross-sectional study; Table S2: Criteria for high-low-medium regarding knowledge, gravity of symptoms; Table S3: Difference between demographics and the respondents knowledge score.

## References

[1] Awine T., Malm K., Bart-Plange C., Silal S.P. (2017). Towards malaria control and elimination in Ghana: Challenges and decision making tools to guide planning. Glob Health Action.

[2] World Health Organization (2017). World Malaria Report 2017.

[3] World Health Organization (2016). World Malaria Report 2015.

[4] World Health Organization (2019). World Malaria Report 2019.

[5] Deku J.G., Lokpo S.Y., Kye-Amoah K.K., Orish V.N., Ussher F.A., Esson J., Aduko R.A., Dakorah M.P., Osei-Yeboah J. (2018). Malaria Burden and Trend among Clients Seeking Healthcare in the Western Region: A 4-Year Retrospective Study at the Sefwi-Wiawso Municipal Hospital, Ghana. Open Microbiol. J..

[6] Ukoli F. (1992). Prevention and Control of Parasitic Diseases in Tropical Africa: The Main Issues.

[7] Godwin J.T., Mbaawuaga M.E., Akaa P.D., Alao O.O., Peters E.J., Utsalo S.J., Okwori E.E., Akosu T.J., Etukumana E.A. (2010). Sociocultural factors influencing the control of malaria in an endemic city in north central Nigeria. Int. J. Biol. Med. Res..

[8] Jegede A.S., Amzat J., Salami K.K., Adejumo P.O., Oyetunde M.O. (2005). Perceived causes of malaria among market women in Ibadan, Nigeria. Afr. J. Psychol. Study Soc. Issues.

[9] Sachs J., Malaney P. (2002). The economic and social burden of malaria. Nature.

[10] Sabin L.L., Rizal A., Brooks M.I., Singh M.P., Tuchman J., Wylie B.J., Joyce K.M., Yeboah-Antwi K., Singh N., Hamer D.H. (2010). Attitudes, knowledge, and practices regarding malaria prevention and treatment among pregnant women in Eastern India. Am. J. Trop. Med. Hyg..

[11] Asante K.P., Abokyi L., Zandoh C., Owusu R., Awini E., Sulemana A., Amenga-Etego S., Adda R., Boahen O., Segbaya S. (2010). Community perceptions of malaria and malaria treatment behaviour in a rural district of Ghana: Implications for artemisinin combination therapy. BMC Public Health.

[12] Assan A., Takian A., Hanafi-Bojd A.A., Rahimiforoushani A., Nematolahi S. (2017). Knowledge, attitude, and practice about malaria: Socio-demographic implications for malaria control in rural Ghana. J. Public Health Policy.

[13] Attu H., Adjei J.K. (2018). Local knowledge and practices towards malaria in an irrigated farming community in Ghana. Malar. J..

[14] Brenyah R.C., Osakunor D.N.M., Ephraim R.K.D. (2013). Factors influencing urban malaria: A comparative study of two communities in the Accra Metropolis. Afr. Health Sci..

[15] Hoffman S.J., Guindon G.E., Lavis J.N., Ndossi G.D., Osei E.J., Sidibe M.F., Boupha B. (2011). Assessing healthcare providers’ knowledge and practices relating to insecticide-treated nets and the prevention of malaria in Ghana, Laos, Senegal and Tanzania. Malar. J..

[16] Nyavor K.D., Kweku M., Agbemafle I., Takramah W., Norman I., Tarkang E., Binka F. (2017). Assessing the ownership, usage and knowledge of insecticide treated nets (ITNs) in malaria prevention in the hohoe municipality, Ghana. Pan Afr. Med. J..

[17] Mbohou Nchetnkou C., Foko L.P.K., Lehman L.G. (2020). Knowledge, Attitude, and Practices towards Malaria among Employees from Enterprises in the Town of Douala, Cameroon. BioMed Res. Int..

[18] Okwa O.O., Soremekun B.M., Adeseko O., Raheem A.M. (2012). Artisans and traders’ knowledge, attitude and practices of malaria in selected areas of Lagos, Nigeria. Mechanics.

[19] Adusei K.A., Owusu-Ofori A. (2018). Prevalence of Plasmodium parasitaemia in blood donors and a survey of the knowledge, attitude and practices of transfusion malaria among health workers in a hospital in Kumasi, Ghana. PLoS ONE.

[20] Buabeng K.O., Matowe L.K., Smith F., Duwiejua M., Enlund H. (2010). Knowledge of medicine outlets’ staff and their practices for prevention and management of malaria in Ghana. Pharm. World Sci..

[21] Owusu-Ofori A., Gadzo D., Bates I. (2016). Transfusion-transmitted malaria: Donor prevalence of parasitaemia and a survey of healthcare workers knowledge and practices in a district hospital in Ghana. Malar. J..

[22] Prah J.K., Yeboah-Sarpong A., Pinkrah R., Ewudzi-Acquah E. (2019). Assessment of the knowledge, attitude and practices of prescribers regarding malaria diagnosis: A cross sectional study among Ghanaian prescribers. Pan Afr. Med. J..

[23] Owusu E.D., Cremers A.L., Brown C.A., Mens P.F., Grobusch M.P. (2018). Knowledge, attitudes and practices regarding malaria in people living with HIV in rural and urban Ghana. Acta Trop..

[24] Fuge T.G., Ayanto S.Y., Gurmamo F.L. (2015). Assessment of knowledge, attitude and practice about malaria and ITNs utilization among pregnant women in Shashogo District, Southern Ethiopia. Malar. J..

[25] Anene-okeke C.G., Isah A., Aluh D.O., Ezeme A.L. (2018). Knowledge and practice of malaria prevention and management among non-medical students of university of Nigeria, Nsukka. Int. J. Community Med. Public Health.

[26] Usman S.O., Ipinmoye T.O., Adu A.S., Fadero T., Edet-Utan O., Isola I.N., Ibrahim A., Oluberu O.A., Ojediran T.E., Akintayo-Usman N.O. (2015). Knowledge and practice of malaria prevention among nonmedical students of higher institutions in Ondo State, Nigeria. Int. J..

[27] Sumari D., Dillip A., Ndume V., Mugasa J.P., Gwakisa P.S. (2016). Knowledge, attitudes and practices on malaria in relation to its transmission among primary school children in Bagamoyo district, Tanzania. Malar. World J..

[28] Habtai H., Ghebremeskel T., Mihreteab S., Mufunda J., Ghebremichael A. (2008). Knowledge, attitudes and practices (KAP) about malaria among people visiting referral hospitals of Eritrea. J. Eritrean Med. Assoc..

[29] Singh R., Musa J., Singh S., Ebere U.V. (2014). Knowledge, attitude and practices on malaria among the rural communities in Aliero, Northern Nigeria. J. Family Med. Prim. Care.

[30] Khairy S., Al-Surimi K., Ali A., Shubily H.M., Al Walaan N., Househ M., El-Metwally A. (2017). Knowledge, attitude and practice about malaria in south-western Saudi Arabia: A household-based cross-sectional survey. J. Infect. Public Health.

[31] Tamirat A., Geremew M., Abamecha F., Wollancho W. (2016). Knowledge, Attitude and Practice about malaria in Maji District, Bench Maji Zone, Southwest Ethiopia. J. Trop. Dis. Public Health.

[32] Forero D.A., Chaparro P.E., Vallejo A.F., Benavides Y., Gutiérrez J.B., Arévalo-Herrera M., Herrera S. (2014). Knowledge, attitudes and practices of malaria in Colombia. Malar. J..

[33] Hlongwana K.W., Mabaso M.L., Kunene S., Govender D., Maharaj R. (2009). Community knowledge, attitudes and practices (KAP) on malaria in Swaziland: A country earmarked for malaria elimination. Malar. J..

[34] Dlamini S.V., Liao C.W., Dlamini Z.H., Siphepho J.S., Cheng P.C., Chuang T.W., Fan C.K. (2017). Knowledge of human social and behavioral factors essential for the success of community malaria control intervention programs: The case of Lomahasha in Swaziland. J. Microbiol. Immunol. Infect.

[35] Ghana Statistical Service-GSS (2020). Ghana Malaria Indicator Survey 2019.

[36] Amusan V.O., Umar Y.A., Vantsawa P.A. (2017). Knowledge, attitudes and practices on malaria prevention and control among private security guards within Kaduna Metropolis, Kaduna State-Nigeria. Sci. J. Public Health.

[37] Munisi D.Z., Nyundo A.A., Mpondo B.C. (2019). Knowledge, attitude and practice towards malaria among symptomatic patients attending Tumbi Referral Hospital: A cross-sectional study. PLoS ONE.

[38] Ismail N.E., Jimam N.S., Dapar M.L. (2019). Assessment of primary health care workers’ knowledge, attitudes and practices on uncomplicated malaria management in Plateau state, Nigeria. Int. J. Pharm. Sci. Res..

[39] Adegun Joel A., Adegboyega J.A., Awosusi Ajoke O. (2011). Knowledge and the preventive strategies of malaria among migrant farmers in Ado-Ekiti Local Government Area of Ekiti State, Nigeria. Am. J. Sci. Ind. Res..

[40] Jimam N.S., Ismail N.E. (2020). Predictors of patients’ knowledge, attitudes and practices (KAP) regarding uncomplicated malaria in the primary healthcare facilities of Plateau state, Nigeria. J. Health Res..

[41] Adedotun A., Morenikeji O., Odaibo A. (2010). Knowledge, attitudes and practices about malaria in an urban community in south-western Nigeria. J. Vector Borne Dis..

[42] World Health Organization (2015). Guidelines for the Treatment of Malaria.

