# Supplementary material for: Unravelling the Perspectives of Day and Night Traders in Selected Markets within a Sub-Saharan African City with a Malaria Knowledge, Attitude and Practice Survey
Source: Int J Environ Res Public Health. 2021 Mar 26;18(7):3468. doi: 10.3390/ijerph18073468 (PMC8037644; doi:10.3390/ijerph18073468)
Supplement: Supplementary file 1 [file ijerph-18-03468-s001.pdf]

Supplementary Material S1

QUESTIONNAIRE

Project Title: **KNOWLEDGE, ATTITUDE, PRACTICES (KAP) ON MALARIA AND PLASMODIUM PARASITE CARRIAGE AMONG DAY AND NIGHT MARKET WORKERS IN GREATER ACCRA, GHANA: A CROSS-SECTIONAL STUDY**

A. SOCIO-ACADEMIC DATA

1. Respondent ID No: ……………… Date:…………/……………/…….……………

2. Gender [ ] Male [ ] Female 3. Age: [ ] 15-20 [ ] 21-25 [ ] 25-30 [ ] > 30

4. Marital Status [ ] Married [ ] Widowed [ ] Divorced [ ] Separated [ ] Never married

5. Name of Market: ………………………………………………………………………………………

6. What do you sell? [ ] Prepared Food [ ] Fruits and Vegetables [ ] Dressings [ ] Drinks
[ ] Electricals and Electronics [ ] Other ………………………………………………………………..

7. Your area of residence………………………………………………………………………………

8. Medium of getting to the market. [ ] Vehicle [ ] Walk [ ] Other…………………………………….

9. How often do you come to the market in a week? [ ] Once [ ] Two times [ ] Three times [ ] Four times [ ] Five times [ ] Six times [ ] Through-out

10. Do you sell at other markets as well? [ ] Yes [ ] No

If Yes please specify where…………………………………………………………….…………….

B. KNOWLEDGE ON MALARIA

11. Have you heard of Malaria? [ ] Yes [ ] No

12. Source of Information [ ] Home (Family, neighbours and friends) [ ] Health workers

[ ] Media (Radio, TV, publications) [ ] Other ……………………………………………………

13. What transmits malaria? [ ] House flies [ ] Ticks [ ] Fleas [ ] Mosquitoes [ ] Cockroaches

14. Malaria cases occur more in which season in Ghana? [ ] Dry [ ] Wet

15. Which category of people is vulnerable? [ ] Fair coloured people [ ] Dark coloured people [ ] Pregnant women and children [ ] Adults

16. Does siting on the sun for long hours cause malaria? [ ] Yes [ ] No

17. What are some symptoms of malaria? (You can tick more than one)
[ ] Vomiting [ ] Weakness [ ] Loss of appetite [ ] High body Temperature

[ ] No idea [ ] Others ………………………………………………………………….
18. What is your understanding of the word “fever”?

[ ] Vomiting [ ] Weakness [ ] Loss of appetite [ ] Feeling hot [ ] Body pains [ ] Chills
[ ] Headache

19. What are some preventive measures you know of? (You can tick more than one)
[ ] Treated bed nets [ ] Using insecticide spray/repellents [ ] Eating hygienic food [ ] Not sitting on the sun for long hours [ ] Getting enough sleep [ ] Exercising

20. Is malaria curable? [ ] Yes [ ] No

21. Do you know of the National Malaria Control program? [ ] yes [ ] No

22. Do you think malaria can be eradicated in Ghana? [ ] Yes [ ] No

23. Is malaria only in Africa? [ ] Yes [ ] No

24. Overall how would you rate your knowledge on malaria
[ ] fair [ ] Good [ ] Very good [ ] Excellent

C: ATTITUDE ON MALARIA

25. Have you ever had malaria? [ ] Yes [ ] No

26. If yes, how many times? [ ] 1-3 times [ ] 4 – 6 times [ ] 7-10 times [ ] More than 10 times

27. What symptoms made you conclude it was malaria?
[ ] Fever [ ] Vomiting [ ] Weakness [ ] Loss of appetite [ ] Anaemia [ ] No idea

[ ] Others ……………………………………………………………………….

28. Do you always take a lab test to confirm malaria? [ ] Yes [ ] No

29. If yes, where do you have your lab test? [ ] Hospital / Clinic [ ] Pharmacy [ ] Private Lab [ ] Home

30. What is the first thing you will do if have the malaria-like symptoms?
[ ] Go to the hospital [ ] Go to the drug store [ ] Self-treat at home [ ] Do nothing

31. What is the most important factor that makes you decide to seek care at the hospital when you have malaria-like symptoms?
[ ] The condition [ ] Cost [ ] Duration of symptoms [ ] Availability of time

32. Has any of your close family members had malaria? [ ] Yes [ ] No

33. Was it in the period you also had malaria? [ ] Yes [ ] No

D: PRACTICES ON MALARIA

34. What is the best treatment of malaria for you?
[ ] ACTs [ ] Chloroqiune [ ] Paracetamol [ ] Other…………………………….……………

35. Which type of medicine do you prefer for malaria treatment?
[ ] Orthodox [ ] Traditional (Herbal)

36. If you prefer herbal medicine, why? [ ] Has no side effect [ ] More effective [ ] Less cost
[ ] Easy access [ ] People recommendation

37. Do you use treated bed net? [ ] Yes [ ] No
If No, why?
[ ] Discomfort [ ] Allergic to content of the net [ ] It makes people get sick [ ] No reason

[ ] Other……………………………………………………………………..……………………

38. Which malaria preventive measures do you use?
[ ] Clearing of bushes and stagnant water [ ] Residual spraying [ ] Use of bed nets
[ ] Insecticide spray / coil [ ] Other………………………………………………………………

39. Why did you prefer a to use such preventive measures?
[ ] Has no side effect [ ] More effective [ ] Less cost [ ] Easy access
[ ] People recommend it

40. Would you volunteer to tell people about malaria? [ ] Yes [ ] No

Supplementary Material S2

Criteria for high-low-medium regarding knowledge, gravity of symptoms

In this study, the respondents' responses to the 12 knowledge questions were used to compute their composite knowledge level – Each question had a score of 1 if the respondent answered correctly and a score of 0 if they responded wrongly. The scores were then categorized into three groups, with those with scores of 50% (6/12) and below considered as having poor knowledge levels; those who's scores ranged from 7 to 9 were considered to have moderate knowledge, and those who scored from 10 to 12 were considered to have high knowledge on malaria.

Our rationale is similar to that used by Prah, J.K., et al., (2019) in their study where prescribers' knowledge on malaria diagnostic tests was assessed using six questions. A correct answer scored 1 and a wrong answer scored 0. A respondent was deemed to have adequate knowledge if he scored more than 50% (at least 4 out of 6).

However, we used a three-point scaling system (poor, moderate and High) as opposed to (adequate and inadequate) so as to capture a true knowledge level.

Prah, J.K., et al., *Assessment of the knowledge, attitude and practices of prescribers regarding malaria diagnosis: a cross sectional study among Ghanaian prescribers.* The Pan African Medical Journal, 2019. **34**.

Supplementary material S3

Difference between demographics and the respondents knowledge score

From the Adjusted ordinal logistic regression model, the proportional odds of having higher knowledge on malaria was not significantly different between day traders and night traders (AOR: 1.23, 95% CI: 0.89–1.70), p = 0.211). (Table S3a)

Compared to traders in the age range 15–20 years, the proportional odds of having higher knowledge on malaria was about 2 times high among traders aged 26–20 years (AOR: 1.90, 95% CI: 1.09–3.28, p = 0.022) and 2.8 times high among traders aged 36-40 years (AOR: 2.79, 95% CI: 1.22–6.38, p = 0.015).

Compared to traders whose homes, family and friends are their source of information on malaria, the proportional odd of higher knowledge on malaria was 2.8 times high among those whose source of information are health workers (AOR: 2.86, 95% CI: 1.74–4.71, $p < 0.001$), and about 2.2 times high for traders whose source of information are from the mass media (AOR: 2.17, 95% CI: 1.44–3.27, $p < 0.001$). The proportional odd of higher knowledge on malaria was 86% less among traders whose source of information on malaria are not either from their homes, family or neighbours, health workers or the mass media (AOR: 0.14, 95% CI: 0.05–0.40, $p < 0.001$). (Table S3a)

The proportional odd of higher knowledge on malaria was 1.72 times high among traders who do not rely on laboratory test for confirmation of malaria suspicion (AOR: 1.72, 95% CI: 1.18–2.50, $p = 0.005$). Also, the proportional odd of higher knowledge on malaria was 63% less among traders prefer herbs for malaria treatment to ACT for malaria treatment (AOR: 0.37, 95% CI: 0.17–0.79, $p = 0.011$).

The proportional odd of higher knowledge on malaria was 1.45 times significantly high for traders who uses insecticide-treated bed nets (AOR: 1.45, 95% CI: 1.02–2.04, $p = 0.036$). (Table S3a)

Table S3a: Ordered logistic regression model of factors associated with higher level of knowledge about malaria

	**Ordinal Logistic Regression Model of Higher Level of Knowledge about Malaria**			
	**Unadjusted models**		**Adjusted model**	
Variables & categories	COR [95% CI]	*p*-Value	AOR [95% CI]	*p*-Value
Type of trader				
Night	1.00 [reference]		1.00 [reference]	
Day	1.17 [0.86-1.57]	0.315	1.23 [0.89-1.70]	0.211
Age Group				
15–20 years	1.00 [reference]		1.00 [reference]	
21–25 years	1.06 [0.63-1.78]	0.840	1.08 [0.61-1.91]	0.801
26–30 years	1.80 [1.10-2.95]	0.020	1.90 [1.09-3.28]	0.022
31–35 years	1.22 [0.73-2.05]	0.440	1.32 [0.75-2.34]	0.336
36–40 years	1.95 [0.93-4.10]	0.077	2.79 [1.22-6.38]	0.015
>40 years	1.14 [0.62-2.12]	0.674	1.11 [0.57-2.18]	0.761
Sources of Information on Malaria				
Home, family and neighbors	1.00 [reference]		1.00 [reference]	
Health workers	2.37 [1.51-3.70]	<0.001	2.86 [1.74-4.71]	<0.001
Mass media (radio, TV, etc.)	1.99 [1.37-2.90]	<0.001	2.17 [1.44-3.27]	<0.001
Other	0.13 [0.05-0.34]	<0.001	0.14 [0.05-0.40]	<0.001
Respondents' Reliance on Laboratory Tests for Confirmation of Malaria Suspicion				
Yes	1.00 [reference]		1.00 [reference]	
No	1.77 [1.27-2.48]	0.001	1.72 [1.18-2.50]	0.005
Respondents' Malaria Episodes Sometimes Coincided with their Friends' Malaria Episodes				
Yes	1.00 [reference]		1.00 [reference]	
No	2.14 [0.99-4.62]	0.052	1.45 [0.65-3.22]	0.364
Respondents' Choice of Drugs for Malaria Treatment				
ACTs	1.00 [reference]		1.00 [reference]	
Chloroquine	0.42 [0.20-0.85]	0.016	0.60 [0.22-1.65]	0.325
Paracetamol	0.83 [0.41-1.65]	0.586	1.18 [0.50-2.78]	0.708
Herbal	0.41 [0.23-0.73]	0.002	0.37 [0.17-0.79]	0.011
Other	0.43 [0.25-0.73]	0.002	0.51 [0.22-1.18]	0.117
Respondents' Preferred Type of Malaria Treatment				
Orthodox	1.00 [reference]		1.00 [reference]	
Herbal	0.53 [0.36-0.78]	0.001	0.93 [0.48-1.81]	0.824
Use of Insecticide-Treated Bed Nets				

Yes	1.58 [1.18-2.11]	0.002	1.45 [1.02-2.04]	0.036
No	1.00 [reference]		1.00 [reference]	
Reason for Chosen Malaria Prevention Measure				
Perceived lack of side effects	1.00 [reference]		1.00 [reference]	
Perceived superior efficiency	0.82 [0.41-1.64]	0.580	0.92 [0.44-1.92]	0.828
Lower cost of choice	0.52 [0.23-1.15]	0.104	0.82 [0.34-1.97]	0.655
Easy access to choice	0.57 [0.28-1.19]	0.136	0.69 [0.31-1.50]	0.347
Owing to recommendations by others	0.57 [0.25-1.28]	0.174	0.68 [0.28-1.62]	0.381

COR: crude odds ratio. AOR: adjusted odds ratio. CI: confidence interval

Table S3b: Ordinal logistics modelling of knowledge score and demographic parameters

	COR (95% CI)	AOR (95% CI)
Age	0.9540874 (0.8629913, 1.0545554)	0.9581126 (0.8510275, 1.078596)
Gender	0.8833798 (0.6563986, 1.1873497)	0.9130268 (0.6675248, 1.247695)
Marital status	1.0267516 0.9631182 1.0945505	1.0073066 (0.9333819, 1.087042)
Market	1.037880 (0.987513, 1.090738)	1.1349175 (0.9762194 1.320624)
Product	1.0487091 (0.9738006, 1.1295714)	1.0349778 (0.9571784, 1.119319)
Residence	1.0119104 (0.9880575, 1.0363680)	0.9557298 (0.8887277, 1.027297)
Time at Market	1.0021944 (0.8946807, 1.1218770)	0.9884670 (0.8803382, 1.108938)

Ordinal logistics modelling of knowledge score and demographic parameters showed no significance in the relationship. Adjusting for other demographic parameters did not reveal any significance either.

Knowledge score (good, moderate, poor)

COR crudes Odds Ratio

AOR adjusted Odds Ratio

95% CI 95% confidence interval
